# Supplementary figures and images for: The PiNe box: Development and validation of an electronic device to time-lock multimodal responses to sensory stimuli in hospitalised infants
Source: PLoS One. 2023 Jul 13;18(7):e0288488. doi: 10.1371/journal.pone.0288488 (PMC10343045; doi:10.1371/journal.pone.0288488)

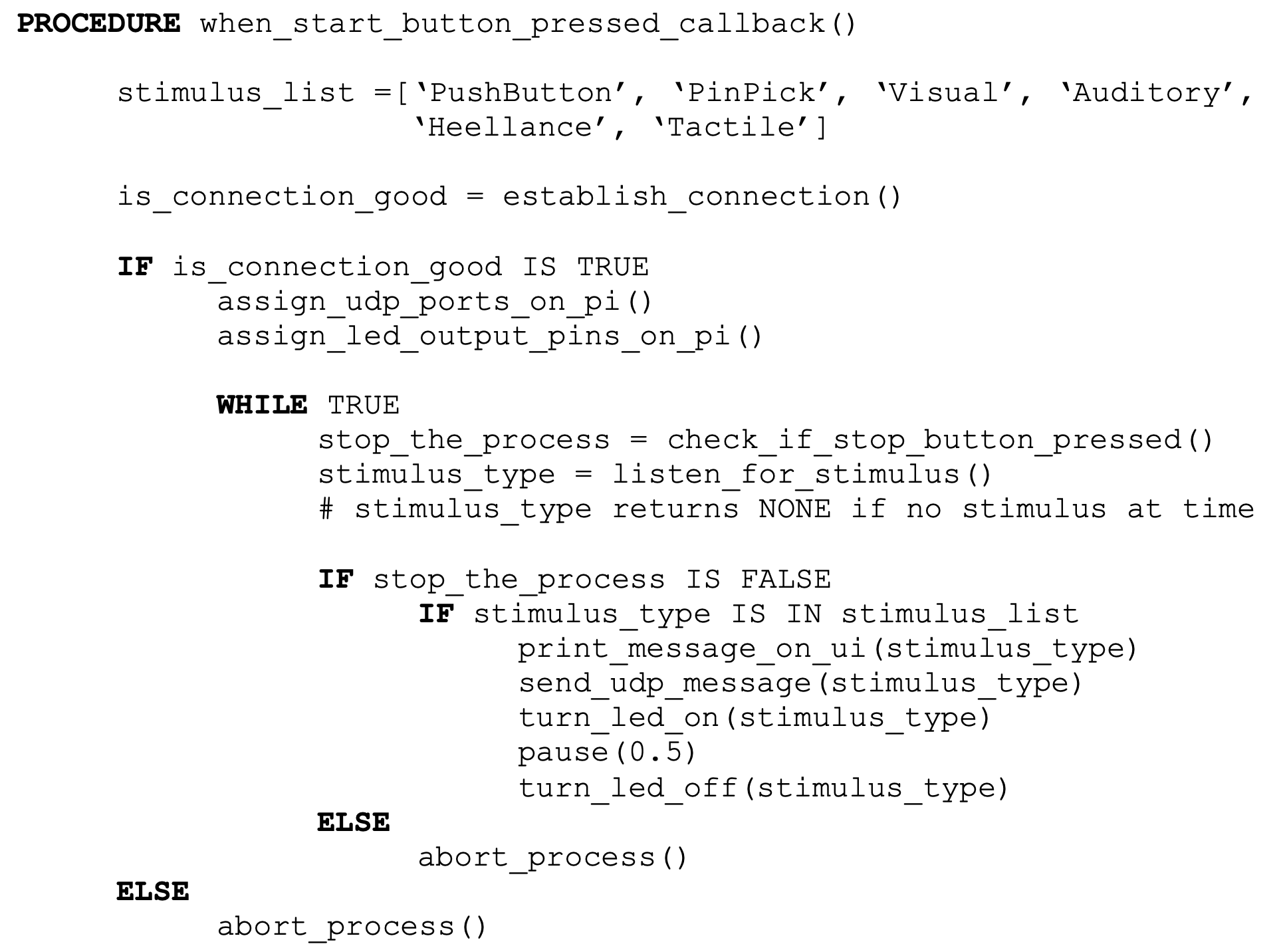

Supplement: S1 Fig — Running the Graphical User Interface, establishing connections, reading of the input stimulus triggers, generation of the UDP (User Datagram Protocol) output messages and transmission to the monitoring laptop, and switching on an LED on the PiNe box following stimulus triggers are all controlled by a bespoke module that is written fully in Python 3. The overall process of the code is summarised in this pseudo code. (PNG) [file pone.0288488.s001.png]

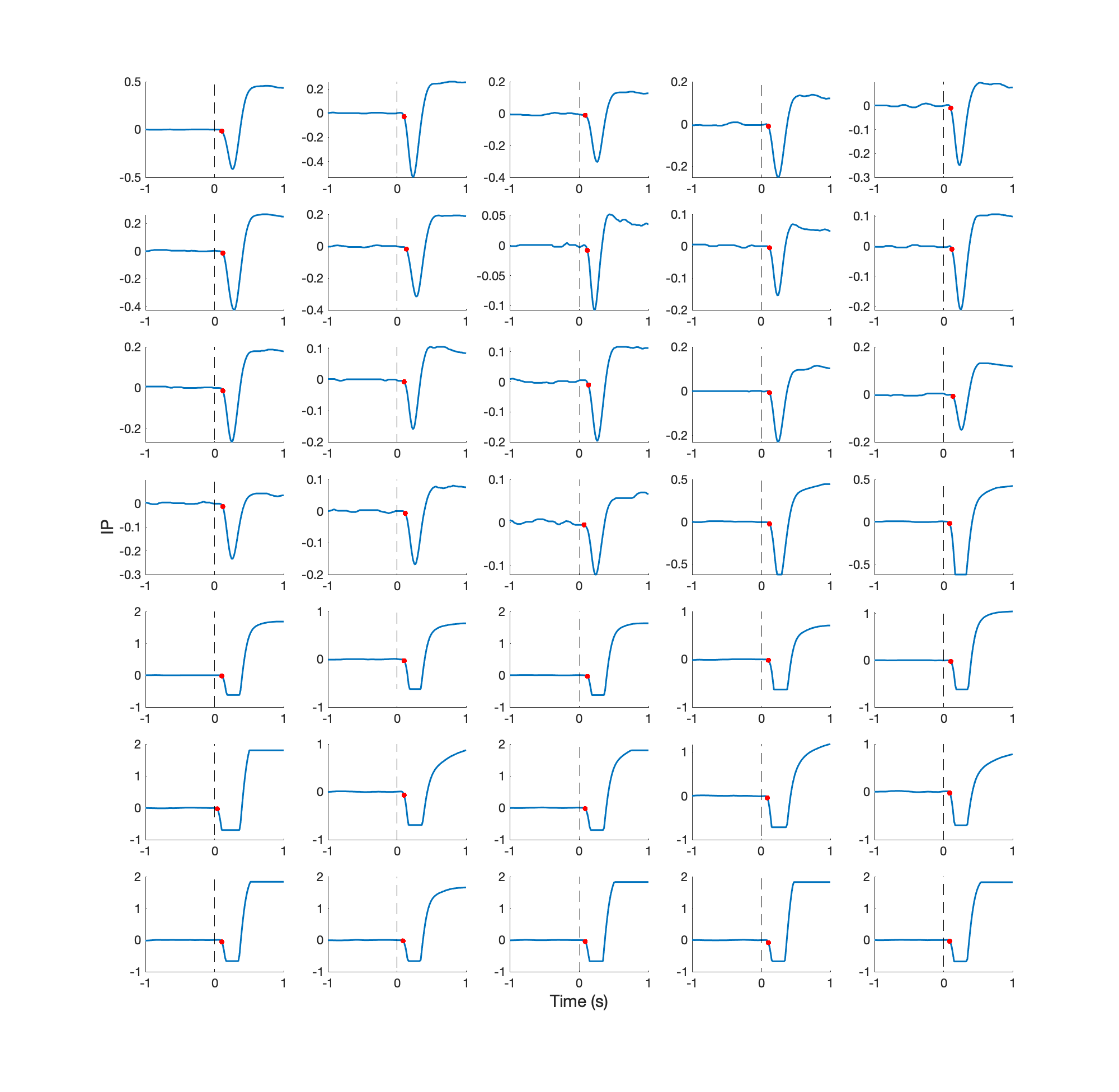

Supplement: S2 Fig — To assess the precision of time-locking of events to the vital signs monitor (Study 2) the push button was pressed onto one of the ECG electrodes during recordings of background noise, generating a visible artefact on the IP (impedance pneumography) signal. The recording during 35 button presses is shown here, with each row a single recording session. The blue lines are the IP signal, time = 0 (black dashed lines) is the time of the event mark annotation from the push button (marked on the recordings via the PiNe box), red dot indicates the start of the artefact generated by the push button calculated as the point at which the signal went below a threshold defined as 3% of the minimum signal value in the 1 second after the event mark. (PNG) [file pone.0288488.s002.png]

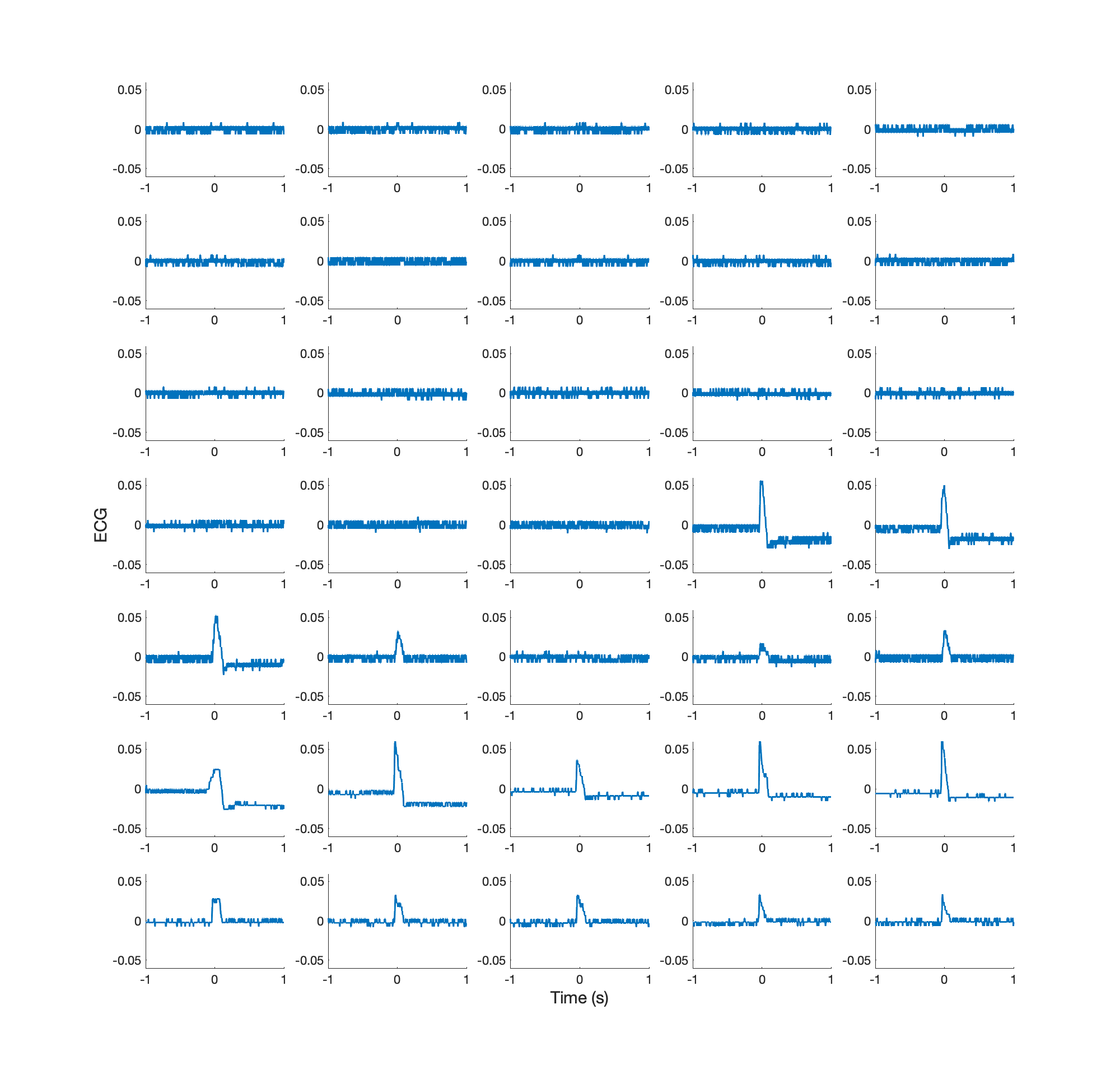

Supplement: S3 Fig — To assess the precision of time-locking of events to the vital signs monitor (Study 2) the push button was pressed onto one of the ECG electrodes during recordings of background noise. Whilst this always generated a visible artefact on the IP (impedance pneumography) signal (S2 Fig), there was only a large visible artefact on the ECG trace on some occasions. This difference is due to the inbuilt filtering of the Philips monitor which is different for the different signals. The recording during 35 button presses is shown here, with each row a single recording session. The blue lines are the ECG signal, time = 0 is the time of the event mark annotation from the push button (marked on the recordings via the PiNe box). (PNG) [file pone.0288488.s003.png]
