# Supplementary material for: The PiNe box: Development and validation of an electronic device to time-lock multimodal responses to sensory stimuli in hospitalised infants
Source: PLoS One. 2023 Jul 13;18(7):e0288488. doi: 10.1371/journal.pone.0288488 (PMC10343045; doi:10.1371/journal.pone.0288488)
Supplement: S1 Table — Values are median (lower quartile, upper quartile) or number (%). PMA = postmenstrual age. Apgar scores were missing from 2 infants. (DOCX) [file pone.0288488.s004.docx]

| PMA (weeks) | 37.7 (35.8, 40.1) |
| --- | --- |
| Gestational age at birth (weeks) | 37.1 (33, 39.9) |
| *Sex*  Female  Male | 16 (31)  35 (69) |
| Birth weight (g) | 2970 (1873, 3555) |
| *Mode of delivery*  Normal vaginal delivery  Assisted vaginal delivery  Elective caesarean section  Emergency caesarean section | 19 (37)  15 (16)  7 (14)  17 (33) |
| *Apgar score*  at 1 minute  at 5 minutes | 9 (7, 10)  10 (10, 10) |

**S1 Table. Infant demographics for Study 2.**

Values are median (lower quartile, upper quartile) or number (%). PMA = postmenstrual age. Apgar scores were missing from 2 infants.
